# Frequencies and Trends of Myocardial Infarction Symptoms From the Years 1985-2019: A Register-based, Real-world Analysis

**DOI:** 10.1016/j.cjco.2025.07.015

**Published:** 2025-07-30

**Authors:** Sophia Wolfermann, Timo Schmitz, Philip Raake, Bernhard Kuch, Jakob Linseisen, Christa Meisinger

**Affiliations:** a Epidemiology, Medical Faculty, University of Augsburg, Augsburg, Germany; b Department of Cardiology, Respiratory Medicine and Intensive Care, University Hospital Augsburg, Augsburg, Germany; c Stiftungskrankenhaus Nördlingen Department of Internal Medicine/Cardiology/Intensive Care, Donau-Ries-Kliniken Nördlingen, Germany

**Keywords:** acute myocardial infarction, atypical symptoms, chest pain, shortness of breath, registry

## Abstract

**Background:**

Our aim in this study was to identify the frequencies of typical and atypical acute myocardial infarction (AMI) symptoms over the past 35 years as well as age and sex differences.

**Methods:**

In this study we used data from the population-based Augsburg Myocardial Infarction Registry. All patients (N = 23,905) 25-74 years of age and hospitalized with AMIs occurring between 1985 and 2019 were included in this analysis. During their hospital stay, patients were interviewed about their symptoms at the acute event and information from patient records was used. Multivariable adjusted logistic regression analyses were conducted to investigate the trends of AMI symptoms over time.

**Results:**

On comparing the time interval 1985-1995 vs 2006-2019, there was a decrease in frequencies (*P* < 0.001 for all) for symptoms of typical chest pain (83.5% vs 80.0%), pain in the left shoulder/arm/hand (52.1% vs 44.9%), pain between the shoulder blades (23.8% vs 19.5%), nausea/vomiting (36.0% vs 30.1%), and fear of death/feeling of annihilation (30.7% vs 14.7%), whereas shortness of breath increased significantly over time (43.8% vs 48.4%, *P* < 0.001). Multivariable logistic regression analysis confirmed the decrease of frequency of AMI symptoms over the past decades. The only exception was occurrence of shortness of breath, where a significantly independent increase was observed when comparing 1985-1995 and 2006-2019 (odds ratio 1.22, 95% confidence interval 1.13-1.32). Atypical symptoms occurred more frequently in older patients and women.

**Conclusions:**

Although there has been a decrease in the frequency of most AMI symptoms over almost 4 decades, AMIs are still commonly accompanied by typical chest pain. In particular, AMI must be considered if shortness of breath is present.

## Résumé

**Contexte:**

Notre objectif dans cette étude était d’examiner la fréquence des symptômes caractéristiques et des symptômes atypiques de l’infarctus aigu du myocarde (IAM) au cours des 35 dernières années, de même que les différences liées à l’âge et au sexe.

**Méthodologie:**

Cette étude a utilisé les données du registre d’Augsburg (Allemagne) sur l’infarctus du myocarde fondé sur la population. Tous les cas de patients hospitalisés en raison d’un IAM survenus entre 1985 et 2019 chez des patients âgés de 25 à 74 ans (N = 23 905) ont été inclus dans l’analyse. Durant leur hospitalisation, on a interrogé les patients sur leurs symptômes liés à l’IAM aigu et on a examiné les renseignements consignés dans leur dossier médical. Des analyses de régression logistique ajustées en fonction de variables multiples ont été menées afin d’évaluer la tendance des symptômes liés à l’IAM au fil du temps.

**Résultats:**

En comparant les données relatives aux intervalles de temps couvrant les années 1985-1995 et 2006-2019, on a observé une baisse de la fréquence (toutes les valeurs *p* < 0,001) des symptômes caractéristiques suivants : douleur thoracique (83,5 % *vs* 80,0 %), douleur dans l’épaule/le bras/la main gauche (52,1 % *vs* 44,9 %), douleur entre les omoplates (23,8 % *vs* 19,5 %), nausées/vomissements (36,0 % *vs* 30,1 %) et peur de la mort/sentiment d’anéantissement (30,7 % *vs* 14,7 %), alors qu’on a noté une augmentation significative de l’incidence d’un essoufflement au fil du temps (43,8 % *vs* 48,4 %; valeur *p* < 0,001). L’analyse de régression logistique multivariée a confirmé une diminution de la fréquence des symptômes de l’IAM au cours des dernières décennies. La seule exception concerne l’occurrence de l’essoufflement, pour lequel une augmentation indépendante significative a été observée entre 1985-1995 et 2006-2019 (rapport de cotes = 1,22; intervalle de confiance à 95 % : 1,13-1,32). Les symptômes atypiques étaient survenus à une fréquence plus élevée chez les patients âgés et les femmes.

**Conclusions:**

Même si on a observé une diminution de la fréquence de la plupart des symptômes d’IAM sur près de quatre décennies, les IAM sont toujours couramment associés à une douleur thoracique caractéristique. Plus particulièrement, le diagnostic d’IAM doit être envisagé en présence d’un essoufflement.

According to the World Health Organization, cardiovascular diseases (CVDs) remain among the most common causes of death.^1^ Currently, around one third of all deaths in Germany are attributable to CVD, and in Europe the CVD mortality is approximately 2.5 times higher for men than for women.^2^ Although in recent years there has been a decrease in CVD mortality due to improved early detection and treatment, myocardial infarction ranks second among the most common causes of death from CVD.^3^ It is well known that, in acute myocardial infarction (AMI), the extent and type of tissue damage is associated with an increased risk of adverse events such as arrhythmia, heart failure, and death.^4^ The development of necrosis follows a temporal pattern, so that approximately 90 minutes after the infarction 80% of the affected area is necrotic and complete necrosis occurs after about 6 hours.^5^

Primary percutaneous coronary intervention is recommended for patients with ST-segment elevation myocardial infarction (STEMI), with guidelines suggesting a first medical contact to device time of ≤ 90 minutes.^6^ A prompt and accurate assessment and interpretation of symptoms is essential to meet this time frame.^7^ Typical symptoms include chest, arm, or jaw pain, alongside a range of other atypical symptoms such as nausea, vomiting, and shortness of breath.^7^ It is assumed that the symptomatology of an AMI differs between women and men, with women expected to exhibit more atypical symptoms.^8^ There are also indications that the leading symptoms of an AMI change depending on age.^9^

In recent decades, greater efforts have been made in cardiovascular prevention with lifestyle changes and preventive medications to reduce incidence and mortality and the cost burden of CVD.^10^ At the same time, educating the public and CVD risk patients about the symptoms of a heart attack and the importance of prompt treatment has also become increasingly relevant.^11^ It remains unclear and has yet to be investigated whether these measures and changes have influenced the types and extent of reported symptoms of a heart attack and whether the symptoms reported have changed over the decades. The extent to which sex- and age-specific differences in symptomatology have emerged in this context has also not been examined. Studies have shown that women and the elderly present with more atypical symptoms of myocardial infarction when compared with men and younger patients, respectively.^8^^,^^12^^,^^13^ Atypical symptoms may contribute to the lower likelihood of a diagnosis and delayed treatment, resulting in poorer outcomes. The purpose of this study was to identify the prevalence of typical symptoms, including chest pain, pain of the left shoulder/arm/hand, or jaw pain, and atypical symptoms, such as pain between shoulder blades or of the upper abdomen, nausea/vomiting, dizziness/vertigo, and shortness of breath, in patients with AMI over the past 4 decades using data from a population-based myocardial infarction registry. In addition, we aimed to determine whether there are age- and sex-specific differences in this regard.

## Methods

### Data collection

The data used in this study were derived from the population-based Augsburg Myocardial Infarction Registry. The registry was implemented in 1984 by the World Health Organization under the name **Moni**toring Trends and Determinants in **Ca**rdiovascular Disease (MONICA), which aimed to completely capture hospitalized AMIs and their associated features such as symptoms, diagnostic procedures, and invasive and noninvasive treatments between the years 1985 and 1995.^14^ From MONICA, the Myocardial Infarction Registry of the Cooperative Health Research in the Augsburg Region (KORA) emerged in 1996, initiated by the Helmholtz Zentrum Munich.^15^ Since 2021, the registry has continued as the Augsburg Myocardial Infarction Registry, based at the University Hospital of Augsburg. All AMIs in individuals 25-74 years of age and who had their primary residency within the study region were included, with the age extended up to 84 years starting in 2009.^16^, ^17^, ^18^

Since October 1985 (until today), all AMI cases in the Augsburg study region have been recorded under effectively the same criteria over the whole study period. The study region of Augsburg, Bavaria, in southern Germany includes the city of Augsburg, the county of Augsburg, and the county of Aichach-Friedberg (approximately 700,000 inhabitants). In this study region, 8 hospitals cooperate closely to ensure comprehensive data collection.

For our analysis, all patients 25-74 years of age and hospitalized with AMIs occurring between 1985 and 2019 were included (23,905 patients). After exclusion of patients with missing data on the symptom of typical chest pain (n = 362), a maximum of 23,543 patients could be included in the study. Patient data were collected by trained study nurses via face-to-face interview using standardized questionnaires during the hospitalization period. To ensure complete data, information from patients’ records was also used. This facilitated the creation of a comprehensive data set at the onset and duration of AMI symptoms as well as diagnostic parameters and information from electrocardiographic and laboratory analyses. The choice of treatment method and treatment course, as well as the various comorbidities and risk factors potentially predisposing to AMI, were also recorded.^19^^,^^20^ Ethics approval for the original data collection was obtained from the Bavarian Medical Association, following the ethical guidelines of the Helsinki Declaration. All patients provided written informed consent for data collection and processing.^15^^,^^20^^,^^21^

### Exposure

The time period 1985-2019 was considered as the exposure period in our analysis. To investigate any association between time period and the prevalence of symptoms, the entire time frame was divided into 3 (roughly equal) intervals based on the year in which the AMI occurred. The first interval covers the years 1985-1995, comprising 5126 cases. In the second interval, from 1996 to 2005, there were 5835 cases and, in the third interval, from 2006 to 2019, there were 12,582 patients included.

### Outcomes

The outcomes of this study included various AMI symptoms. During the face-to-face interview, patients were questioned about their symptoms in the context of the acute event and could respond with “yes” or “no.” Several typical symptoms possibly occurring during an AMI were queried, including the presence of chest pain and radiation of pain to the left or right arm, shoulder, or hand, as well as pain between the shoulder blades. In addition, atypical symptoms were recorded, such as upper abdominal pain, jaw pain, sweating, nausea and vomiting, shortness of breath, and a feeling of annihilation/fear of death. Typical chest pain symptoms were defined according to the MONICA project as definite if sudden onset of chest pain (defined as pain or a feeling of pressure or tightness behind the breastbone) was > 20 minutes.

### Statistical analysis

#### Primary analysis

Characteristics of the study sample and frequencies of each symptom were calculated for all 3 time intervals. Subsequently, χ^2^ tests were conducted to test for differences between the intervals. Logistic regression analyses were conducted with the different time intervals as the independent variable and the respective symptoms as the outcome variable. The interval 1985-1995 was used as the reference interval. Based on prior literature, the logistic regression models were adjusted for the following confounders, which may influence the exposure, the outcome, or both^22^, ^23^, ^24^: infarction type (STEMI, non–ST-segment elevation myocardial infarction [NSTEMI], or bundle branch block), age, sex (man, woman), smoking status (current smoker, ex-smoker, never smoker), diabetes mellitus (yes, no), first AMI or reinfarction, and type of treatment. Two symptom variables, “dizziness” and “loss of consciousness,” were excluded from the logistic regression analyses because information on these symptoms was not available for the whole study period.

### Secondary analyses

In additional descriptive analyses, the frequencies of symptoms by time interval were also determined separately for the different age groups and for men and women. To investigate differences between younger and older AMI patients in terms of acute symptoms over the period studied, patients were divided into 3 age groups: 25-54, 55-64, and 65-74 years. Because the rate of AMI rises with age, the first age group had to cover a wider age range of 30 years, which ensured enough cases within this group. Differences between the time intervals were also tested using χ^2^ tests. All analyses were performed using SPSS version 29.0.1 (IBM Corp, Armonk, NY). Reporting followed the **St**rengthening the **R**eporting of **Ob**servation Studies in **E**pidemiology (STROBE) statement checklist.^25^

## Results

The characteristics of the study sample for the 3 time intervals are presented in Table 1. In comparing the time intervals 1985-1995 vs 2006-2019, the proportion of patients with hypertension (54.9% vs 79.5%) and diabetes (23.6% vs 35.0%) increased significantly over time (*P* < 0.001), whereas the proportion of current smokers decreased (35.4% vs 29.1%, *P* < 0.001). Furthermore, the proportions of NSTEMI patients (46.7% vs 55.2%) and patients receiving reperfusion therapy (36.6% vs 81.9%) increased over time (*P* < 0.001).

**Table 1:** Description of the characteristics of patients with AMI (25-74 years) by time interval

Characteristics	1985-1995	1996-2005	2006-2019	*P* value
Sex (men)	3878 (74.2%)	4499 (76.2%)	9090 (71.2%)	< 0.001
Age 25-54 years	1284 (24.6%)	1411 (23.9%)	2322 (25.3%)	< 0.001
Age 55-64 years	1751 (33.5%)	1964 (33.2%)	2791 (30.4%)	
Age 65-74 years	2190 (41.9%)	2533 (42.9%)	4073 (44.3%)	
Incident infarction (yes)	4075 (78.5%)	4698 (79.8%)	10,153 (79.6%)	0.212
NSTEMI	2270 (46.7%)	2900 (51.4%)	6676 (55.2%)	< 0.001
STEMI	2148 (44.2%)	2344 (41.5%)	4171 (34.5%)	
BBB	443 (9.1%)	400 (7.1%)	1256 (10.4%)	
Hypertension (yes)	2827 (54.9%)	4219 (71.7%)	10,146 (79.5%)	< 0.001
Hyperlipidemia (yes)	2858 (58.0%)	3057 (52.1%)	4523 (35.4%)	0.307
Diabetes (yes)	1222 (23.6%)	1816 (30.8%)	4464 (35.0%)	< 0.001
Current smoker	1849 (35.4%)	1900 (32.2%)	3711 (29.1%)	< 0.001
Ex-smoker	1388 (26.6%)	1708 (28.9%)	3938 (30.8%)	
Never smoker	1536 (29.4%)	1610 (27.3%)	3796 (29.7%)	
No information	452 (8.7%)	690 (11.7%)	2469 (10.4%)	
Reperfusion therapy (yes)	1890 (36.6%)	4195 (71.5%)	10,418 (81.9%)	< 0.001

Data expressed as number (%).

AMI, acute myocardial infarction; BBB, bundle branch block; NSTEMI, non–ST-segment elevation myocardial infarction; STEMI, ST-segment elevation myocardial infarction.

Table 2 shows the frequencies (in number and percentage of patients) of the different AMI symptoms for the total sample by time intervals. In all 3 time intervals, typical chest pain was the most frequently reported symptom, followed by the also common symptoms of pain in the left shoulder/arm/hand, sweating, and shortness of breath. Almost all AMI symptoms differed significantly (*P* < 0.05) between the 1985-1995 and 2006-2019 periods, with decreases in proportions of patients reporting typical chest pain (83.5% vs 80.0%), pain in the left shoulder/arm/hand (52.1% vs 44.9%), pain in the right shoulder/arm/hand (27.1% vs 24.9%), pain between the shoulder blades (23.8% vs 19.5%), nausea/vomiting (36.0% vs 30.1%), and fear of death/feeling of annihilation (30.7% vs 14.7%). Shortness of breath was the only symptom to increase significantly over the study period (43.8% vs 48.4%, *P* < 0.001). No significant differences could be observed between time intervals for the symptoms of sweating and pain in the throat/jaw. The frequency of the symptom dizziness/vertigo increased, and the frequency of the symptom syncope/unconsciousness decreased from the second to the third time interval (true only for these time intervals, because both symptoms were not assessed completely during the first time interval).

**Table 2:** Presenting symptoms of the total sample of patients aged 25-74 years by time interval

Symptom	1985-1995	1996-2005	2006-2019	*P* value
Typical chest pain (yes)	4279 (83.5%)	4895 (83.9%)	7246 (80.0%)	< 0.001
Pain in left shoulder/arm/hand (yes)	2660 (52.1%)	2777 (47.1%)	4097 (44.9%)	< 0.001
Pain in right shoulder/arm/hand (yes)	1370 (27.1%)	1498 (25.4%)	2268 (24.9%)	0.013
Pain between shoulder blades (yes)	1198 (23.8%)	1272 (21.6%)	1775 (19.5%)	< 0.001
Pain in upper abdomen (yes)	640 (12.7%)	521 (8.8%)	970 (10.7%)	< 0.001
Pain in throat/jaw (yes)	1230 (24.4%)	1335 (22.7%)	2086 (22.9%)	0.057
Sweating (yes)	2474 (48.8%)	2834 (48.1%)	4308 (47.3%)	0.183
Nausea/vomiting (yes)	1834 (36.0%)	1822 (30.9%)	2749 (30.1%)	< 0.001
Shortness of breath (yes)	2252 (43.8%)	2550 (43.2%)	4426 (48.4%)	< 0.001
Dizziness/vertigo (yes)	4 (0.9%)	651 (13.8%)	1748 (19.2%)	< 0.001
Syncope/unconsciousness (yes)	4 (0.9%)	344 (7.3%)	457 (5.0%)	< 0.001
Fear of death/feeling of annihilation (yes)	1547 (30.7%)	1267 (21.5%)	1343 (14.7%)	< 0.001

Data expressed as number (%).

### Logistic regression analyses

Multivariable logistic regression analyses on the total sample showed an inverse relationship for the second and third time interval compared with the first time interval and the reported symptoms, except for the symptom shortness of breath (Table 3). The lowest odds was found for the symptom of fear of death/feeling of annihilation (odds ratio 0.35, 95% confidence interval 0.32-0.38) when comparing the third vs the first time interval. In contrast, a significantly higher odds was observed for the third time interval vs the first interval for shortness of breath (odds ratio 1.22, 95% confidence interval 1.13-1.23).

**Table 3:** Associations between different time periods (exposure; reference period 1985 to 1995) and specific symptoms (outcome) in the age group 25-74 years

Symptom (outcome)	1996-2005		2006-2019	
	OR (95% CI)	*P* value	OR (95% CI)	*P* value
Typical chest pain	0.85 (0.76-0.95)	0.005	0.54 (0.48-0.60)	< 0.001
Pain in left shoulder/arm/hand	0.75 (0.69-0.81)	< 0.001	0.62 (0.57-0.67)	< 0.001
Pain in right shoulder/arm/hand	0.87 (0.80-0.96)	0.004	0.78 (0.72-0.86)	< 0.001
Pain between shoulder blades	0.87 (0.79-0.96)	0.004	0.71 (0.64-0.78)	< 0.001
Pain in upper abdomen	0.68 (0.60-0.77)	< 0.001	0.82 (0.73-0.92)	0.001
Pain in throat/jaw	0.86 (0.78-0.94)	0.002	0.79 (0.72-0.87)	< 0.001
Sweating	0.90 (0.82-0.97)	0.009	0.76 (0.70-0.83)	< 0.001
Nausea/vomiting	0.79 (0.73-0.86)	< 0.001	0.74 (0.68-0.80)	< 0.001
Shortness of breath	1.02 (0.94-1.11)	0.626	1.22 (1.13-1.32)	< 0.001
Fear of death/feeling of annihilation	0.61 (0.55-0.67)	< 0.001	0.35 (0.32-0.38)	< 0.001

Results presented are from the multivariable adjusted analyses. A logistic regression model was calculated for each individual symptom as an outcome. Data were adjusted for infarction type, age, sex, smoking status, diabetes mellitus, first acute myocardial infarction or reinfarction, and type of treatment.

CI, confidence interval; OR, odds ratio.

### Results of secondary analyses

There was a downward trend over time for the symptoms of typical chest pain, pain in the left shoulder/arm/hand, and pain in the throat/jaw among those 55-64 and 65-74 years of age (Supplemental Tables S1, S2, and S3). In contrast, such a trend was not observed in the younger age group (25-54 years), where these symptoms tended to develop in the opposite direction (Supplemental Table S1). Regarding shortness of breath, an increase was noted for all age groups over the years. Across all age groups, dizziness/vertigo was observed with increased frequency over the entire period, particularly in the 2 younger age groups.

Typical chest pain was the most frequently reported symptom for both men and women (Supplemental Tables S4 and S5), yet there were no significant differences between the time intervals for women (Supplemental Table S5); on the other hand, men showed a noticeable decrease in frequency of this symptom (Supplemental Table S4). The frequency of the symptom pain in the left shoulder/arm/hand decreased significantly in men and women. In contrast, shortness of breath increased in both sexes over time, with a more pronounced increase in women. The symptom dizziness/vertigo became more frequent over time and was reported less frequently in men than in women. The frequency of upper abdominal pain increased over time in women, but it decreased in men. Furthermore, over time, women were more likely than men to experience vomiting, nausea, and pain between the shoulder blades.

## Discussion

Our results suggest that between 1985 and 2019 there were changes in the frequency of reporting of typical and atypical symptoms associated with AMI. There was a decrease in frequencies for the symptoms of typical chest pain, pain of the left shoulder/arm/hand, pain between shoulder blades, nausea/vomiting, and fear of death/feeling of annihilation, whereas shortness of breath increased over time (43.8% vs 48.4%, *P* < 0.001). In additional analyses, these findings were confirmed for both sexes and largely also for the individual age groups. Furthermore, atypical symptoms occur more frequently in older patients and women with AMI. Typical chest pain was consistently observed and most frequently occurring symptom over the whole time period in the entire sample and among both men and women and in all age groups. Other studies have also reached the conclusion that chest pain is the main symptom in both sexes with AMI.^26^^,^^27^ In addition, more atypical symptoms are reported by women, which occur with greater variety compared with men.^27^, ^28^, ^29^, ^30^, ^31^ In our study, women with AMI also suffered more frequently from symptoms such as pain between the shoulder blades, nausea/vomiting, throat/jaw pain, and shortness of breath when compared with men. Although the frequency of these atypical symptoms decreased over time in both sexes (except for shortness of breath, which increased over time in both men and women), the sex differences remained. Possible explanations for these differences include different pain tolerance in men and women, differing pathophysiologic mechanisms in development of the AMI, and pre-existing comorbidities.^8^ Women with AMI more frequently than men have diabetes mellitus, hypertension, heart and kidney diseases, and depression.^8^^,^^32^ The INTERHEART study, a large international case-control study, assessed the importance of risk factors for coronary heart disease and the strength of associations between various risk factors and AMI in the entire study population, stratified by geographic region, ethnicity, gender, and age. It was shown that the risk factors classified in the INTERHEART study have a significantly greater impact on the development of AMI in women than in men.^32^^,^^33^ Instead of the primary cause, the atherosclerotic plaque–induced AMI, the pathophysiology in women may be altered; microvascular changes and comorbidities may increase the risk of a type 2 AMI, which is caused by a mismatch between oxygen supply and demand, thereby triggering atypical symptoms.^8^^,^^34^

Some studies have shown that older patients less frequently have typical chest pain compared with younger patients,^35^^,^^36^ as confirmed our study. Furthermore, we observed that other classic AMI symptoms, such as left shoulder/arm/hand pain, as well as atypical symptoms (eg, right shoulder/arm/hand pain) were reported less frequently among older patients in all 3 time intervals. In accordance with prior investigations, shortness of breath was more frequently present in older than in younger patients with AMI.^37^^,^^38^

The data from the Augsburg Myocardial Infarction Registry show that the frequency of almost all AMI symptoms declined significantly over time, independent of infarction type, age, sex, smoking status, diabetes mellitus, first or recurrent AMI, and type of treatment. However, it is notable that shortness of breath increased significantly during the study period. A possible explanation could be that patients today are older when experiencing their AMI and are more likely to suffer from comorbidities such as hypertension and diabetes mellitus.^39^ This can lead to a distorted perception and an interpretation of symptoms when the origin of the complaints is no longer clearly identifiable. In contrast, multimorbidity or existing heart failure can more frequently lead to shortness of breath in the context of an AMI. It is essential to pay more attention to the symptom of dyspnea, as studies have shown that the occurrence of shortness of breath is associated with poorer short- and long-term survival outcomes.^17^^,^^40^

Since the introduction of highly sensitive cardiac troponin I or T diagnostics, minor myocardial infarctions associated with mild symptoms have been diagnosed more frequently. This may be another explanation for the decrease in most typical and atypical AMI symptoms. Furthermore, compared with the 1980s and 1990s, today's faster and better (pain) medications (immediate administration of morphine, aspirin, etc) in cases of an AMI may mean that pain is not perceived as strongly and less clearly remembered at the time of interview during hospitalization.^41^

One could assume that the general population is better informed now than in the past about typical and atypical symptoms that can occur in the context of an AMI. A recent systematic review of the awareness of AMI symptoms revealed a moderate to good knowledge of “classic” symptoms and insufficient knowledge of less obvious symptoms of AMI.^42^ This lack of knowledge about atypical symptoms and the resulting lack of attribution of symptoms to an AMI---which may be more common in older age groups---could be a factor in the higher mortality seen in older patients with AMI.^42^ A survey conducted in the United States in 1998 as part of the **R**apid **E**arly **A**ction for **C**oronary **T**reatment (REACT) study evaluated how many of the 11 symptoms listed as typical heart attack symptoms were perceived by respondents as a sign of their heart attack.^43^^,^^44^ On average, patients experienced only 3 of 11 symptoms, with chest pain being mentioned most frequently. According to the authors, this result can be attributed to the previous lower level of public knowledge about the diverse manifestations of a heart attack, where only typical chest pain was widely known.^43^ Similarly, a street survey conducted in Birmingham, UK in 2009 showed that the public is inadequately informed about the variety of heart attack symptoms, especially people of lower socioeconomic status. The most frequently mentioned symptom was central chest pain, followed by arm pain and shortness of breath.^45^

### Study limitations

There are some limitations to this study. We included only patients 25-74 years of age living in the Augsburg region, and mainly of German nationality. Therefore, the results cannot be applied to older age groups and may not be generalizable to other regions or all ethnic groups. Another limitation may be that not all symptoms that could occur in connection with an AMI were recorded and therefore no statements can be made about other possible symptoms. A further limitation is that the symptoms were self-reported by the patients and recorded days after the acute event and were therefore highly subjective. Also, the possibility of recall bias due to medical history cannot be ruled out. Symptoms in the most severely ill patients, namely those who died within the first few days, could not be recorded due to the lack of an interview; more “severe” symptoms may have been expected in this patient group. The lack of data on cardiac markers and the changing definition of AMI over time driven by using different assays is a further shortcoming of the study. The data collected by the registry do not tie into the fourth universal definition of myocardial infarction.^46^ Unfortunately, renal function (or chronic kidney disease) data are not available for the period 1985-2005, so we could not include that information in the multivariable regression models. Furthermore, we could not distinguish whether the bundle branch blocks were long-standing or new. The electrocardiograms at hospital admission were assessed by a physician, but the Sgarbossa criteria were not applied. Although the regression models were adjusted for several confounders, unmeasured or residual confounding cannot not be entirely excluded.

## Conclusions

Although there has been a decrease in the frequency of most AMI symptoms over at least 3 decades, acute heart attacks are still commonly accompanied by typical chest pain. This applies to both sexes and to all age groups. Most symptoms became less frequent in AMI patients over the years, but it is still important to note that atypical symptoms occur more often in women and with advancing age. The frequency of shortness of breath increased in recent decades and therefore must be considered as a potential angina equivalent. This also must be considered in the context of population-based awareness campaigns on AMI symptoms.
