# Supplementary material for: Frequencies and Trends of Myocardial Infarction Symptoms From the Years 1985-2019: A Register-based, Real-world Analysis
Source: CJC Open. 2025 Jul 30;7(11):1474–81. doi: 10.1016/j.cjco.2025.07.015 (PMC12713181; doi:10.1016/j.cjco.2025.07.015)
Supplement: Supplemental Material [file mmc1.docx]

**Frequencies and trends of myocardial infarction symptoms from the year 1985 to 2019: a register-based real-world analysis**

Sophia Wolfermann, Timo Schmitz, Philip Raake, Bernhard Kuch, Jakob Linseisen, Christa Meisinger

**Supplementary material**

**Supplemental Table S1:** Presenting symptoms in patients **aged 25 to 54 years** (n, %) by time interval

| **Symptom** | 1985 to 1995 | 1996 to 2005 | 2006 to 2019 | p-value* |
| --- | --- | --- | --- | --- |
| Typical chest pain† (yes) | 1072 (83.5%) | 1229 (87.1%) | 1985 (85.5%) | 0.041 |
| Pain left shoulder/arm/hand (yes) | 708 (55.1%) | 775 (54.9%) | 1327 (57.1%) | 0.283 |
| Pain right shoulder/arm/hand (yes) | 397 (30.9%) | 437 (31.0%) | 733 (31.6%) | 0.865 |
| Pain between shoulder blades (yes) | 287 (22.4%) | 295 (20.9%) | 533 (23.0%) | 0.245 |
| Pain upper abdomen (yes) | 174 (13.6%) | 135 (9.6%) | 242 (10.4%) | <0.001 |
| Pain throat/jaw (yes) | 309 (24.1%) | 374 (26.5%) | 658 (28.3%) | 0.043 |
| Sweating (yes) | 709 (55.2%) | 826 (58.5%) | 1337 (57.6%) | 0.452 |
| Nausea/vomiting (yes) | 456 (35.5%) | 485 (34.4%) | 776 (33.4%) | 0.307 |
| Shortness of breath (yes) | 517 (40.3%) | 582 (41.2%) | 1052 (45.3%) | 0.006 |
| Dizziness/vertigo (yes) | 0 (0%) | 178 (12.6%) | 534 (23.0%) | <0.001 |
| Syncope/unconsciousness (yes) | 1 (0.1%) | 81 (5.7%) | 90 (3.9%) | <0.001 |
| Fear of death/feeling of annihilation (yes) | 396 (30.8%) | 390 (27.6%) | 442 (19.0%) | <0.001 |

*Chi^2^-test

†sudden onset of chest pain (defined as pain or a feeling of pressure or tightness behind the breastbone) lasting longer than 20 minutes

**Supplemental Table S2:** Presenting symptoms in patients **aged 55 to 64 years** (n, %) by time interval

| **Symptom** | 1985 to 1995 | 1996 to 2005 | 2006 to 2019 | p-value* |
| --- | --- | --- | --- | --- |
| Typical chest pain† (yes) | 1457 (83.2%) | 1641 (83.6%) | 2203 (78.9%) | <0.001 |
| Pain left shoulder/arm/hand (yes) | 925 (52.8%) | 955 (48.6%) | 1255 (45.0%) | <0.001 |
| Pain right shoulder/arm/hand (yes) | 496 (28.3%) | 517 (26.3%) | 714 (25.6%) | 0.042 |
| Pain between shoulder blades (yes) | 407 (23.2%) | 438 (22.3%) | 547 (19.6%) | 0.003 |
| Pain upper abdomen (yes) | 212 (12.1%) | 185 (9.4%) | 299 (10.7%) | 0.014 |
| Pain throat/jaw (yes) | 432 (24.7%) | 452 (23.0%) | 663 (23.8%) | 0.276 |
| Sweating (yes) | 858 (49.0%) | 969 (49.3%) | 1376 (49.3%) | 0.890 |
| Nausea/vomiting (yes) | 626 (35.8%) | 612 (31.2%) | 868 (31.1%) | <0.001 |
| Shortness of breath (yes) | 741 (42.3%) | 803 (40.9%) | 1351 (48.4%) | <0.001 |
| Dizziness/vertigo (yes) | 173 (9.9%) | 224 (11.4%) | 549 (19.7%) | <0.001 |
| Syncope/unconsciousness (yes) | 3 (0.2%) | 107 (5.4%) | 135 (4.8%) | 0.005 |
| Fear of death/feeling of annihilation (yes) | 559 (31.9%) | 423 (21.5%) | 419 (15.0%) | <0.001 |

*Chi^2^-test

†sudden onset of chest pain (defined as pain or a feeling of pressure or tightness behind the breastbone) lasting longer than 20 minutes

**Supplemental Table S3:** Presenting symptoms in patients **aged 65 to 74 years** (n, %) by time interval

| **Symptom** | 1985 to 1995 | 1996 to 2005 | 2006 to 2019 | p-value* |
| --- | --- | --- | --- | --- |
| Typical chest pain† (yes) | 1750 (79.9%) | 2025 (79.9%) | 3058 (75.1%) | <0.001 |
| Pain left shoulder/arm/hand (yes) | 1027 (46.9%) | 1047 (41.3%) | 1515 (37.2%) | <0.001 |
| Pain right shoulder/arm/hand (yes) | 477 (21.8%) | 544 (21.5%) | 821 (20.2%) | 0.078 |
| Pain between shoulder blades (yes) | 504 (23.0%) | 539 (21.3%) | 695 (17.1%) | <0.001 |
| Pain upper abdomen (yes) | 254 (11.6%) | 201 (7.9%) | 429 (10.5%) | <0.001 |
| Pain throat/jaw (yes) | 489 (22.3%) | 509 (20.1%) | 765 (18.8%) | <0.001 |
| Sweating (yes) | 907 (41.4%) | 1039 (41.0%) | 1595 (39.2%) | 0.018 |
| Nausea/vomiting (yes) | 752 (34.3%) | 725 (28.6%) | 1105 (27.1%) | <0.001 |
| Shortness of breath (yes) | 994 (45.4%) | 1165 (46.0%) | 2023 (49.7%) | 0.003 |
| Dizziness/vertigo (yes) | 0 (0%) | 249 (9.8%) | 665 (16.3%) | <0.001 |
| Syncope/unconsciousness (yes) | 0 (0%) | 156 (6.2%) | 232 (5.7%) | 0.007 |
| Fear of death/feeling of annihilation (yes) | 592 (27.0%) | 454 (17.9%) | 482 (11.8%) | <0.001 |

*Chi^2^-test

†sudden onset of chest pain (defined as pain or a feeling of pressure or tightness behind the breastbone) lasting longer than 20 minutes

**Supplemental Table S4:** Presenting symptoms in **men** **aged 25 to 74 years** (n, %) by time interval

| **Symptom** | 1985 to 1995 | 1996 to 2005 | 2006 to 2019 | p-value* |
| --- | --- | --- | --- | --- |
| Typical chest pain † (yes) | Yes: 3217 (84.4%) | 3741 (84.1%) | 5505 (80.0%) | <0.001 |
| Pain left shoulder/arm/hand (yes) | Yes: 1939 (51.0%) | 2056 (45.8%) | 3009 (43.5%) | <0.001 |
| Pain right shoulder/arm/hand (yes) | Yes: 1026 (27.2%) | 1121 (25.0%) | 1686 (24.4%) | 0.004 |
| Pain between shoulder blades (yes) | Yes: 753 (20.1%) | 823 (18.3%) | 1147 (16.6%) | <0.001 |
| Pain upper abdomen (yes) | Yes: 503 (13.4%) | 387 (8.6%) | 676 (9.8%) | <0.001 |
| Pain throat/jaw (yes) | Yes: 874 (23.3%) | 944 (21.0%) | 1453 (21.0%) | 0.013 |
| Sweating (yes) | Yes: 1926 (51.0%) | 2165 (48.2%) | 3306 (47.7%) | 0.004 |
| Nausea/vomiting (yes) | Yes: 1241 (32.8%) | 1242 (27.7%) | 1857 (26,6%) | <0.001 |
| Shortness of breath (yes) | Yes: 1631 (42.7%) | 1833 (40.8%) | 3249 (46.8%) | <0.001 |
| Dizziness/vertigo (yes) | Yes: 3 (0.8%) | 459 (12.8%) | 1262 (18.2%) | <0.001 |
| Syncope/unconsciousness (yes) | Yes: 4 (1.1%) | 255 (7.1%) | 323 (4.7%) | <0.001 |
| Fear of death/feeling of annihilation (yes) | Yes: 1125 (30.0%) | 855 (19.1%) | 919 (13.3%) | <0.001 |

*Chi^2^-test

†sudden onset of chest pain (defined as pain or a feeling of pressure or tightness behind the breastbone) lasting longer than 20 minutes

**Supplemental Table S5:** Presenting symptoms in **women** **aged 25 to 74 years** (n, %) by time interval

| **Symptom** | 1985 to 1995 | 1996 to 2005 | 2006 to 2019 | p-value* |
| --- | --- | --- | --- | --- |
| Typical chest pain† (yes) | Yes: 1062 (80.8%) | 1154 (83.1%) | 1741 (79.9%) | 0.059 |
| Pain left shoulder/arm/hand (yes) | Yes: 721 (55.3%) | 721 (51.3%) | 1088 (49.6%) | 0.005 |
| Pain right shoulder/arm/hand (yes) | Yes: 344 (26.8%) | 377 (26.8%) | 582 (26.6%) | 0.983 |
| Pain between shoulder blades (yes) | Yes: 445 (34.8%) | 449 (32.0%) | 628 (28.7%) | <0.001 |
| Pain upper abdomen (yes) | Yes: 137 (10.7%) | 134 (9.5%) | 294 (13.4%) | <0.001 |
| Pain throat/jaw (yes) | Yes: 356 (27.8%) | 391 (27.8%) | 633 (28.9%) | 0.696 |
| Sweating (yes) | Yes: 548 (42.4%) | 669 (47.5%) | 1002 (45.8%) | 0.026 |
| Nausea/vomiting (yes) | Yes: 593 (45.3%) | 580 (41.2%) | 892 (40.6%) | 0.020 |
| Shortness of breath (yes) | Yes: 621 (47.0%) | 717 (50.9%) | 1177 (53.5%) | <0.001 |
| Dizziness/vertigo (yes) | Yes: 1 (1.0%) | 192 (17.1%) | 486 (22.2%) | <0.001 |
| Syncope/unconsciousness (yes) | Yes: 0 (0%) | 89 (7.9%) | 134 (6.1%) | 0.057 |
| Fear of death/feeling of annihilation (yes) | Yes: 422 (32.9%) | 412 (29.3%) | 424 (19.4%) | <0.001 |

*Chi^2^-test

†sudden onset of chest pain (defined as pain or a feeling of pressure or tightness behind the breastbone) lasting longer than 20 minutes
